# Interaction between pharmaceutical companies and physicians who prescribe antiretroviral drugs for treating AIDS: Interação entre empresas farmacêuticas e médicos que prescrevem antirretrovirais para o tratamento da aids

**DOI:** 10.1590/1516-3180.2014.1321609

**Published:** 2014-02-01

**Authors:** Mário César Scheffer

**Affiliations:** MSc, PhD. Professor, Department of Preventive Medicine, Faculdade de Medicina da Universidade de São Paulo (FMUSP), São Paulo, Brazil

**Keywords:** Drug industry, Drug prescriptions, Ethics, medical, Anti-retroviral agents, Acquired immunodeficiency syndrome, Indústria farmacêutica, Prescrições de medicamentos, Ética médica, Antirretrovirais, Síndrome de imunodeficiência adquirida

## Abstract

**CONTEXT AND OBJECTIVE:**

Given that Brazil has a universal public policy for supplying medications to treat HIV and AIDS, the aim here was to describe the forms of relationship between physicians and the pharmaceutical companies that produce antiretrovirals (ARVs).

**DESIGN AND SETTING:**

Cross-sectional epidemiological study conducted in the state of São Paulo.

**METHODS:**

: Secondary database linkage was used, with structured interviews conducted by telephone among a sample group of 300 physicians representing 2,361 professionals who care for patients with HIV and AIDS.

**RESULTS:**

: Around two thirds (64%) of the physicians prescribing ARVs for HIV and AIDS treatment in the state of São Paulo who were interviewed declared that they had some form of relationship with pharmaceutical companies, of which the most frequent were receipt of publications (54%), visits by sales promoters (51%) and receipt of small-value objects (47%).

**CONCLUSIONS:**

Two forms of relationship between the pharmaceutical industry and physicians who deal with HIV and AIDS can be highlighted: facilitation of professionals' access to continuing education; and antiretroviral drug brand name promotion.

## Abstract (pt)

**CONTEXTO E OBJETIVO:**

Diante da existência no Brasil de uma política pública universal de fornecimento de medicamentos para tratamento do HIV e aids, o objetivo é descrever formas de relacionamento entre os médicos e as empresas farmacêuticas produtoras de antirretrovirais (ARVs).

**TIPO DE ESTUDO E LOCAL:**

Estudo epidemiológico de tipo transversal realizado no estado de São Paulo.

**MÉTODOS:**

Foi realizado cruzamento entre bancos de dados secundários e entrevistas estruturadas, por meio telefônico, em amostra de 300 médicos representativa de 2.361 profissionais que assistem pacientes com HIV e aids.

**RESULTADOS:**

Cerca de dois terços (64%) dos médicos entrevistados que prescrevem ARVs para tratamento de HIV e aids no Estado de São Paulo declararam que tiveram alguma relação com empresas farmacêuticas, sendo mais frequentes o recebimento de publicações (54%), de visita de propagandistas (51%) e de objetos de pequeno valor (47%).

**CONCLUSÃO:**

Destacam-se duas formas de relacionamento entre a indústria farmacêutica e os médicos que trabalham com HIV-aids: facilitação do acesso dos profissionais à educação continuada e promoção da marca de medicamentos antirretrovirais.

## INTRODUCTION

The AIDS epidemic introduced new elements into the world of drug research and development. It gave rise to unprecedented behavior within the pharmaceutical industry, the fields of medicine and science, the ethics of human research and the organization of services and community mobilization to ensure access to antiretroviral therapy*.* ^1^

In the early 1990s, monotherapy with antiretrovirals (ARVs), followed by combinations of two medications, offered patients modest and ephemeral benefits before the disease evolved. The therapeutic approach to AIDS made unquestionable progress only after the introduction, in 1995, of highly active antiretroviral therapy (HAART), which featured protease inhibitor drugs, thus making ARV combinations more powerful and effective. The role of ARV drugs is to inhibit viral replication, restore the immune system of infected people and reduce occurrences of opportunistic infections and other morbidities.^2^

The pharmaceutical companies responsible for discovering and launching ARVs form one of the most competitive sectors of the global market, dominated by multinational corporations. They are large companies capable of financing and incorporating into their products the main advances that are seen to be possible within the biomedical, biological and chemical sciences.^3^

The main multinationals involved in ARV production in the world have subsidiaries in Brazil. ARVs have been introduced into Brazil in line with the companies' global strategies, local demand and the Ministry of Health's purchasing capacity. The goal of a subsidiary of a foreign pharmaceutical company is to expand the national market of a particular pharmaceutical specialty in order to increase the demand for the drugs developed and produced by the parent company.^4^

Of the 21 ARVs distributed by the Ministry of Health in 2010, 10 were produced by Brazilian pharmaceutical companies in the form of generic drugs. However, foreign companies with patented brands consume most of the public funds earmarked for contracts for purchasing ARVs in Brazil. Approximately 200,000 individuals were undergoing treatment with ARVs within the public healthcare network in Brazil in 2010, and 35,000 new cases of HIV infection are identified per year on average. This is generating a progressive increase in the number of people who will begin to receive ARVs. The Ministry of Health is responsible for recording, purchasing, distributing and preparing clinical guidelines for use of ARVs. These 21 medications, which are in five therapeutic classes, are available through the Brazilian National Health System (Sistema Ъnico de Saъde, SUS), and are dispensed in 677 public pharmacies and healthcare services.^5^

The high consumption of ARVs in Brazil, which are included within a public policy of universal access, means that pharmaceutical companies have set in motion a wide range of promotion strategies and informational and persuasion-related activities, with the objective of encouraging prescription, dispensing and purchasing by the government, and use of their medications.

In this regard, physicians prescribing ARVs, who rely on help from clinical guidelines produced by the government program, but also enjoy professional autonomy at the time of making prescriptions, become the number one target of company marketing.

## OBJECTIVES

The present article had the aim of sizing up and describing the main forms of relationship between physicians who prescribe ARVs and the pharmaceutical industry. At the same time, it sought to address the promotion strategies of the companies producing these medications that form part of the Brazilian public policy of universal access to HIV and AIDS treatment.

## METHOD

This study used a research database that drew up the profile of physicians who prescribe ARVs in the state of Sгo Paulo,^6^ conducted in conjunction with the Department of Preventive Medicine of the University of Sгo Paulo School of Medicine (FMUSP). For the initial study, the population was composed of 2,361 physicians who treated people with HIV in the state of Sгo Paulo and who prescribed antiretroviral drugs between October 2007 and May 2009. The list of prescribing physicians was extracted from the Medication Logistics Control System (SICLOM) of the STD, AIDS and Viral Hepatitis Department of the Ministry of Health. The information about sociodemographic and academic background characteristics was obtained from the physician roster of the Regional Medical Council of the State of Sгo Paulo (Conselho Regional de Medicina do Estado de Sгo Paulo, CREMESP).

In its second phase, the study gathered the opinions of ARV prescribers, in a selected sample representing the complete group of 2,361 physicians. Drawing on approximation to the binomial distribution for normal distribution, with the aim of estimating the parameter π = 0.50 (proportion of largest variance) with a sampling error of 0.06 and a confidence interval of 95%, the number of participants required was found to be approximately n = 300. The selection was made proportionally to the following strata: volume of patients, length of professional experience and region where the physician lived.

The interviews covered job satisfaction, education, experience, training, working conditions, pay, relationship with patients, opinion about the Brazilian anti-AIDS program, and other points. One of the topics addressed, which is the subject of the present article, was the physicians' relationship with the pharmaceutical companies that produce ARVs.

The geographical limitation of the study to the state of Sгo Paulo was established because this is the state that concentrates the highest number of notified cases of AIDS in Brazil, totaling 207,077 from 1980 to June 2011.^7^ This state also contains all of the Medication Dispensing Units within SUS that are integrated into SICLOM, an information system that makes it possible to obtain data on the prescribing physicians.

## RESULTS

Observation of the profile of ARV-prescribing physicians in the state of Sгo Paulo who answered the questionnaire revealed that 48% were male and 52% female; the majority were aged between 24 and 40 years (57%), averaging 39 years; most lived in the metropolitan region of Sгo Paulo (57%), with 52% in the state capital; and 50.6% had completed and 49.4% had not completed their medical residency. Regarding academic background, 30.7% had specialized in infectious diseases; 35.1% had undergone specialized training in other areas and 34.2% did not have any type of specialized training. In this study, specialized training was taken to mean obtainment of a title issued by a medical specialty society, or completion of medicine residency or a master's or doctoral program. Most of the physicians (55%) had up to nine years of experience with HIV and AIDS and, at the time of the survey, 48% were caring for more than 20 patients each. On average, the physicians cared for six patients with HIV and AIDS per day, whereas 75% cared for between one and ten patients on a daily basis. Out of the total number sampled, 11% declared that they were not regular prescribers, since they had seldom prescribed ARVs.

**Figure 1:**
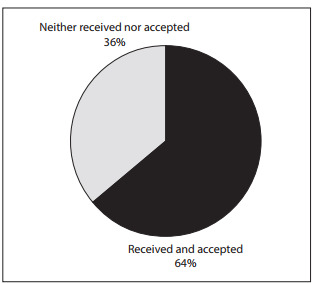
Distribution of physicians who prescribed antiretroviral drugs in the state of São Paulo, according to receipt or non-receipt of benefits from pharmaceutical laboratories, 2010.

Among the doctors who cared for patients with HIV and AIDS in the state of Sгo Paulo, 64% declared that they had received products, benefits or payments from the pharmaceutical laboratories that produce ARVs (Figure 1).

**Figure 2:**
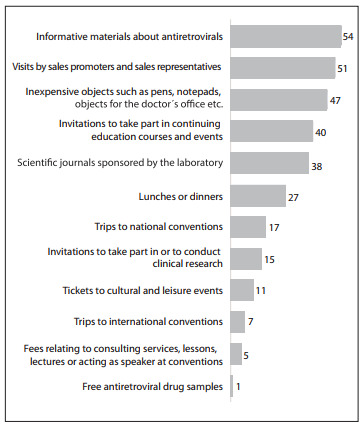
Distribution of physicians who prescribed antiretroviral drugs in the state of São Paulo, according to types of benefits received from pharmaceutical laboratories, 2010.

The most common benefits received (Figure 2) were: informative materials about ARVs (54%); visits by sales promoters and sales representatives (51%); inexpensive objects for the doctor's office (47%); invitations to take part in continuing education courses and events (40%); and scientific journals sponsored by the laboratories (38%).

In smaller proportions, the physicians declared the following benefits: lunches or dinners (27%); trips to national conventions (17%) and international conventions (7%); invitations to take part in or to conduct clinical research (15%); tickets to cultural and leisure events (11%); fees relating to consulting services, lessons, lectures or speaking at conventions (5%); and free ARV drug samples (1%).

With regard to the benefits offered, infectologists (or specialists in infectious diseases) were the physicians who most received small-value objects (p < 0.001) and trips to national conventions (p = 0.021). This statistical significance was obtained through contingency table analysis using the chi-square test (x^2)^ and Fisher's exact test, with the significance level of α < 0.05.^8^ In relation to other benefits, although high numbers were received by the infectologists, statistical significance was not observed (Annex).

**Figure 3:**
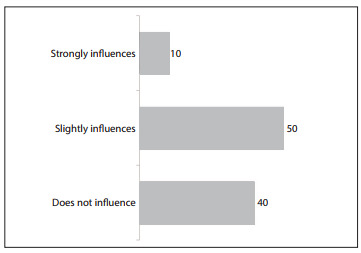
Distribution of physicians who prescribed antiretroviral drugs in the state of São Paulo, according to their opinion regarding the influence of pharmaceutical companies on prescriptions, 2010.

Asked about the laboratories' level of influence on professional activities, most of the interviewees stated that the pharmaceutical companies' actions had a strong influence (10%), slight influence (50%) or no influence (40%) on their prescribing of antiretrovirals (Figure 3).

## DISCUSSION

This study shows that one of the strategies of the ARV industry is to invest in training and information for physicians, which includes sponsorship of courses and production of informative material. Another initiative is financial support for physicians' participation in scientific events and congresses, where the pharmaceutical companies take part in scheduling and organizing satellite events, erect stands and develop promotional activities.

However, it is worth emphasizing that this study shows that only a minority of the physicians who were prescribing ARVs (10%) affirmed that the companies exerted considerable influence over them. 

Although the percentage of physicians who prescribed ARVs (64%) and had received some benefit from the industry was lower than the level that has been identified in other studies evaluating the relationship between physicians and the industry. In the state of Sгo Paulo, 93% of physicians in general were found to have received some benefit from pharmaceutical, equipment, orthosis and prosthesis companies.^9^ In the United States, 94% of physicians were found to have some connection with drug manufacturers.^10^

Some factors may be linked to the relatively low level of interaction between ARV prescribers and the industry. In Brazil, these medications are not traded on the market: they form part of a public program of free distribution through SUS and are included in clinical guidelines that are updated periodically and accepted by the medical community. Another important factor is that some ARVs are already produced in Brazil, in the form of generic drugs, which diminishes the interest in commercial promotion of these medications among national producing laboratories.

On the other hand, ARV use depends on medical prescription. Several of them compete for the same therapeutic indication and new products of patented brands are constantly being launched, thus causing producing companies to resort to all the available resources to conquer the market.

Several variables have been correlated with the act of medical prescribing: technical capacity, intelligence, skills, common sense, motivation, standards of judgment, knowledge accumulation, clinical experience, time dedicated to refresher courses, level of confidence in the sales promoters of the pharmaceutical companies, specialization level, time since graduation, workplace and coexistence with colleagues.^11^

Medical literature, scientific congresses, information on pharmaceutical companies, professional interactions between physicians, continuing education programs and clinical guidelines have an influence on prescribing of medications. Physicians mainly evoke the scientific literature as the greatest influence on prescriptions, but in practice they can also be influenced by the discourse of the pharmaceutical industry.^12^

The marketing practices of pharmaceutical companies are often subtle and indirect. They consist of amicable pressures and games of influence that can go unnoticed, since they are an integral part of the culture of the drug market. Physicians and the drug industry are bound by mutual necessity.^13^ Nonetheless, targeted promotion may possibly create a favorable environment, of sympathy for and receptivity towards the medication.

In Brazil, initiatives targeting improvement of regulations relating to interactions between prescribers and the pharmaceutical industry are still taking shape. 

The Medical Ethics Code (updated in 2010) and Resolutions from the Federal Medical Council and the National Health Surveillance Agency may not be sufficient. Likewise, self-regulatory codes of ethics within the current pharmaceutical market, with manuals on advertising and marketing conduct implemented by drug companies, comprise initiatives that may need to be enhanced in order to ensure greater transparency and higher ethical standards in relationships between producers and prescribers. 

Furthermore, it can be assumed that there is a considerable imbalance in financial resources between the funds made available by the pharmaceutical industry for promotional information relating to ARVs and the funds from the government and professional associations intended for information addressed to physicians. 

The issue of the influence of the industry on prescription choice should form the subject of future studies, considering that much evidence showing that pharmaceutical companies' actions may arouse conflict of interests and may influence physicians' decisions has been presented in the literature.^14^

## CONCLUSION

Two activities can be highlighted regarding the forms of relationship between physicians who prescribe ARVs and pharmaceutical companies: continuing education initiatives (brochures, scientific journals, courses and participation in medical conferences) and drug brand name promotion (sales representative visits and brand-orientated gifts and objects). It was not possible to determine the influence of the relationship involved in physicians' choice of ARVs for prescription. 
